# The comorbidity profiles and medication issues of patients with multiple system atrophy: a systematic cross-sectional analysis

**DOI:** 10.1007/s00415-024-12207-5

**Published:** 2024-02-14

**Authors:** Lan Ye, Stephan Greten, Florian Wegner, Johanna Doll-Lee, Lea Krey, Johanne Heine, Florin Gandor, Annemarie Vogel, Luise Berger, Doreen Gruber, Johannes Levin, Sabrina Katzdobler, Oliver Peters, Eman Dashti, Josef Priller, Eike Jakob Spruth, Andrea A. Kühn, Patricia Krause, Annika Spottke, Anja Schneider, Aline Beyle, Okka Kimmich, Markus Donix, Robert Haussmann, Moritz Brandt, Elisabeth Dinter, Jens Wiltfang, Björn H. Schott, Inga Zerr, Mathias Bähr, Katharina Buerger, Daniel Janowitz, Robert Perneczky, Boris-Stephan Rauchmann, Endy Weidinger, Emrah Düzel, Wenzel Glanz, Stefan Teipel, Ingo Kilimann, Isabel Wurster, Kathrin Brockmann, Daniel C. Hoffmann, Thomas Klockgether, Olaf Krause, Johannes Heck, PROMESA study group, Sylvia Maaß, Madeleine Schubert, Armin Giese, Wolfgang H. Oertel, Werner Poewe, Claudia Trenkwalder, Gregor K. Wenning, Ulrich Mansmann, Martin Südmeyer, Karla Eggert, Brit Mollenhauer, Axel Lipp, Matthias Löhle, Joseph Classen, Alexander Münchau, Jan Kassubek, Daniela Berg, Silvia Egert-Schwender, Cornelia Eberhardt, Friedemann Paul, Kai Bötzel, Birgit Ertl-Wagner, Hans-Jürgen Huppertz, Ingrid Ricard, Elisabeth André, Christiane Blankenstein, Monica Canelo, Marco Düring, Jens Ebentheuer, Christopher Fricke, Alexander Gerbes, Stefan Groiss, Christian Hartmann, Thomas Kirchner, Daniel Kroneberg, Martin Kunz, Stefan Lorenzl, Alexia Moldovan, Anna Noda, Heidi Pape, Gesine Respondek, Eva Schäffer, Alfons Schnitzler, Walter Schulz-Schaeffer, Johannes Schwarz, Cornelia Skowronek, Alexander Storch, Vera Tadic, Dávid Vadász, Benno Zimmermann, Martina Schneider, Günter U. Höglinger, Martin Klietz

**Affiliations:** 1 Department of Neurology, Hannover Medical School, Carl-Neuberg-Str. 1, 30625 Hannover, Germany; 2 Neurologisches Fachkrankenhaus für Bewegungsstörungen/Parkinson, Kliniken Beelitz, 14547 Beelitz-Heilstätten, Germany; 3 Department of Neurology, University Hospital of Munich, Ludwig-Maximilians-Universität (LMU) Munich, Munich, Germany; 4 German Center for Neurodegenerative Diseases (DZNE), Munich, Germany; 5 Munich Cluster for Systems Neurology (SyNergy) Munich, Munich, Germany; 6 German Center for Neurodegenerative Diseases (DZNE), Berlin, Germany; 7 Department of Psychiatry and Psychotherapy, Charité, Universitätsmedizin Berlin, Berlin, Germany; 8 Department of Neurology, Charité-Universitätsmedizin Berlin, Berlin, Germany; 9 Department of Psychiatry and Psychotherapy, Klinikum Rechts der Isar, Technical University Munich, Munich, Germany; 10 Movement Disorder and Neuromodulation Unit, Department of Neurology, Charité, University Medicine Berlin, Charité, Berlin, Germany; 11 German Center for Neurodegenerative Diseases (DZNE), Bonn, Germany; 12 Department of Neurology, University Hospital Bonn, Bonn, Germany; 13 Department of Neurodegenerative Diseases and Geriatric Psychiatry, University Hospital Bonn, Bonn, Germany; 14 German Center for Neurodegenerative Diseases (DZNE), Dresden, Germany; 15 Department of Psychiatry and Psychotherapy, University Hospital Carl Gustav Carus, Technische Universität Dresden, Dresden, Germany; 16 Department of Neurology, University Hospital Carl Gustav Carus, Technische Universität Dresden, Dresden, Germany; 17 German Center for Neurodegenerative Diseases (DZNE), Goettingen, Germany; 18 Department of Psychiatry and Psychotherapy, University Medical Center Goettingen, University of Goettingen, Göttingen, Germany; 19 Neurosciences and Signaling Group, Department of Medical Sciences, Institute of Biomedicine (iBiMED), University of Aveiro, Aveiro, Portugal; 20 Department of Neurology, University Medical Center, Georg August University, Göttingen, Germany; 21 Institute for Stroke and Dementia Research, University Hospital, LMU Munich, Munich, Germany; 22 Department of Psychiatry and Psychotherapy, University Hospital, LMU Munich, Munich, Germany; 23 Ageing Epidemiology Research Unit, School of Public Health, Imperial College London, London, UK; 24 German Center for Neurodegenerative Diseases (DZNE), Magdeburg, Germany; 25 Institute of Cognitive Neurology and Dementia Research, Otto-Von-Guericke University, Magdeburg, Germany; 26 Institute of Cognitive Neuroscience, University College London, London, UK; 27 Clinic for Neurology, Medical Faculty, University Hospital Magdeburg, Magdeburg, Germany; 28 German Center for Neurodegenerative Diseases (DZNE), Rostock-Greifswald, Germany; 29 Department of Psychosomatic Medicine, Rostock University Medical Center, Rostock, Germany; 30 German Center for Neurodegenerative Diseases (DZNE), Tübingen, Germany; 31 Department of Neurodegenerative Diseases, Hertie Institute for Clinical Brain Research, University of Tübingen, Tübingen, Germany; 32 DIAKOVERE Henriettenstift and Department of General Medicine and Palliative Care, Center for Medicine of the Elderly, Hannover Medical School, Carl-Neuberg-Straße 1, 30625 Hannover, Germany; 33 Center for Geriatric Medicine, Hospital DIAKOVERE Henriettenstift, Schwe-Mannstrasse 19, 30559 Hannover, Germany; 34 Institute for Clinical Pharmacology, Hannover Medical School, Carl-Neuberg-Straße 1, 30625 Hannover, Germany

**Keywords:** Multiple system atrophy, Comorbidities, Polypharmacy, Genitourinary system diseases, Drug-drug interactions

## Abstract

**Background:**

Multiple system atrophy (MSA) is a complex and fatal neurodegenerative movement disorder. Understanding the comorbidities and drug therapy is crucial for MSA patients’ safety and management.

**Objectives:**

To investigate the pattern of comorbidities and aspects of drug therapy in MSA patients.

**Methods:**

Cross-sectional data of MSA patients according to Gilman et al. (2008) diagnostic criteria and control patients without neurodegenerative diseases (non-ND) were collected from German, multicenter cohorts. The prevalence of comorbidities according to WHO ICD-10 classification and drugs administered according to WHO ATC system were analyzed. Potential drug-drug interactions were identified using AiDKlinik®.

**Results:**

The analysis included 254 MSA and 363 age- and sex-matched non-ND control patients. MSA patients exhibited a significantly higher burden of comorbidities, in particular diseases of the genitourinary system. Also, more medications were prescribed MSA patients, resulting in a higher prevalence of polypharmacy. Importantly, the risk of potential drug-drug interactions, including severe interactions and contraindicated combinations, was elevated in MSA patients. When comparing MSA-P and MSA-C subtypes, MSA-P patients suffered more frequently from diseases of the genitourinary system and diseases of the musculoskeletal system and connective tissue.

**Conclusions:**

MSA patients face a substantial burden of comorbidities, notably in the genitourinary system. This, coupled with increased polypharmacy and potential drug interactions, highlights the complexity of managing MSA patients. Clinicians should carefully consider these factors when devising treatment strategies for MSA patients.

## Introduction

Multiple system atrophy (MSA) is a progressive neurodegenerative disease that impacts the nigrostriatal system, cerebellum, pons, inferior olives, key brainstem and spinal cord nuclei involved in autonomic function [1, 2]. Clinically, its manifestation is characterized by a comprehensive array of symptoms. These include motor symptoms such as parkinsonism, dystonia, cerebellar ataxia, dysphagia, dysarthria, as well as autonomic failure involving neurogenic orthostatic hypotension, supine hypertension, urge incontinence, nocturia, incomplete bladder emptying, sexual dysfunction and constipation. Additionally, a range of other non-motor symptoms is present, including rapid eye movement sleep behavior disorder, sleep apnea, nocturnal stridor, depression and pain [3]. Depending on the presenting motor phenotype, MSA can be further categorized into parkinsonian (MSA-P) and cerebellar phenotype (MSA-C) [2]. It was shown that MSA progresses rapidly, leading to severe disability within 5–6 years and death within 10 years of symptom onset [4–7]. However, the comorbidity profiles and medication issues in MSA patients have not been studied in detail yet. This gap in research is of utmost significance. Multimorbidity is associated with increased mortality, impaired quality of life and heightened utilization of the healthcare resources [8]. Given the multifaceted nature of MSA, patients often require a variety of medications to manage their symptoms. This complexity is compounded by the presence of comorbidities, resulting in intricate medication regimens. Polypharmacy, which is defined as the routine use of five or more medications according to a report from the Word Health Organization (WHO), was also reported to be associated with numerous negative clinical outcomes such as frailty, hospitalization and even higher mortality [9]. Investigating the situation of multimorbidity and polypharmacy are vital for providing comprehensive, safe and effective medical care.

In this study, we examined comprehensively the medical histories of 254 MSA patients from multiple centers across Germany. Our analysis focused on identifying the comorbidities most prevalent in MSA as well as specific to distinct MSA phenotypes, and on investigating the complex landscape of drug interactions. Through these efforts, our intent was to contribute to the advancement of patient care and the development of effective management strategies for individuals afflicted by MSA, ultimately aiming to elevate their quality of life.

## Methods

### Participants

Ethical approval was obtained from the local Ethics Committee at Hannover Medical School and all participating study centers. Cross-sectional data of 254 MSA patients were collected in multiple German centers with a special expertise in movement disorders (e.g. Hannover, Beelitz-Heilstätten, and Munich). A part of the MSA patient data acquired originated from the PROMESA study [10]. The MSA diagnosis and phenotype was determined by a movement disorder specialist according to diagnostic criteria for MSA [2]. The data of 363 control patients without neurodegenerative diseases (non-ND) from the German, multicenter cohort study DANCER were used as a comparison group (German Center for Neurodegenerative Diseases, DZNE). Relatives of patients with neurological diseases, interested persons and neurological patients without neurodegenerative disease were participating in this DANCER study. Participants did not receive any financial compensation for participating in the study.

### Data acquisition

An experienced movement disorder specialist in all participating centers performed the survey and examination. Demographic information (age, sex, and symptom onset), clinical scores and medical history (comorbidities and medication) were collected from patients or their caregivers. Data from the most recent visit were used for analysis. The comorbidities were classified according to the first and second level of the World Health Organization (WHO) International Classification of Diseases, 10th Revision (WHO ICD-10, latest version, 2019). The medication was classified according to the annually updated WHO Anatomical Therapeutic Chemical (ATC) system (https://www.whocc.no/atc_ddd_index/). The levodopa equivalent dose (LED) was calculated according to the report from Schade et al. [11].

Potential drug-drug interactions (pDDIs) were identified using the well-established clinical decision support system (CDSS) AiDKlinik® (AID, version 01.05.2020; Dosing GmbH, Heidelberg, Germany). The analysis did not include whether these pDDIs resulted in actual side effects. PDDIs were differentiated according to their severity ranging from “disputed evidence”, “light interaction”, “moderate interaction”, and “severe interaction” to “contraindicated combination”.

### Statistical analysis

All statistical analyses in the present study were performed using R commander. Continuous variables were reported as mean and standard deviation (± SD). The unpaired t-test was carried out to compare continuous variables for normally distributed data and the Mann–Whitney U test was employed for comparing continuous variables in cases of non-normally distributed data. The chi-squared test was used to compare proportions for categorical variables.

## Results

### Patient characteristics

Table 1 displays the demographic characteristics of the study participants. The MSA group comprised of 254 patients, while the non-ND control group included 363 individuals. The mean age of MSA patients was 63.8 ± 8.5 years, while the mean age of the matched control group was 63.7 ± 13.7 years (*p* > 0.05).

**Table 1:** Main demographic and clinical characteristics of MSA and non-ND patients

Characteristic	MSA (n = 254)	Non-ND (n = 363)
Age, mean ± SD (min, max)	63.75 ± 8.52 (46.82)	63.69 ± 13.69 (20.91)
Sex, female (%)	125 (49.2)	202 (55.6)
Geriatric patients, n (%)	40 (15.75)	22 (6.06)
Age ≥ 70, n (%)	72 (28.35) ***	148 (40.77)
Multimorbidity, n (%)	165 (64.96)**	191 (52.62)
Polypharmacy, n (%)	165 (64.96)***	62 (17.08)

Geriatric patients are those with age ≥ 70 years, multimorbidity and polypharmacy

*MSA* Multiple system atrophy, *SD* Standard deviation, *non-ND* without neurodegenerative diseases

^*^p < 0.05, **p < 0.01, ***p < 0.001, chi-squared test

Regarding sex distribution, the MSA group had 125 females, accounting for 49.2% of the group, while the control group had 202 females, making up 55.6% of the group. Although the proportion of females was slightly higher in the control group, the difference was not statistically significant (*p* > 0.05).

### Comorbidities

**Fig. 1:**
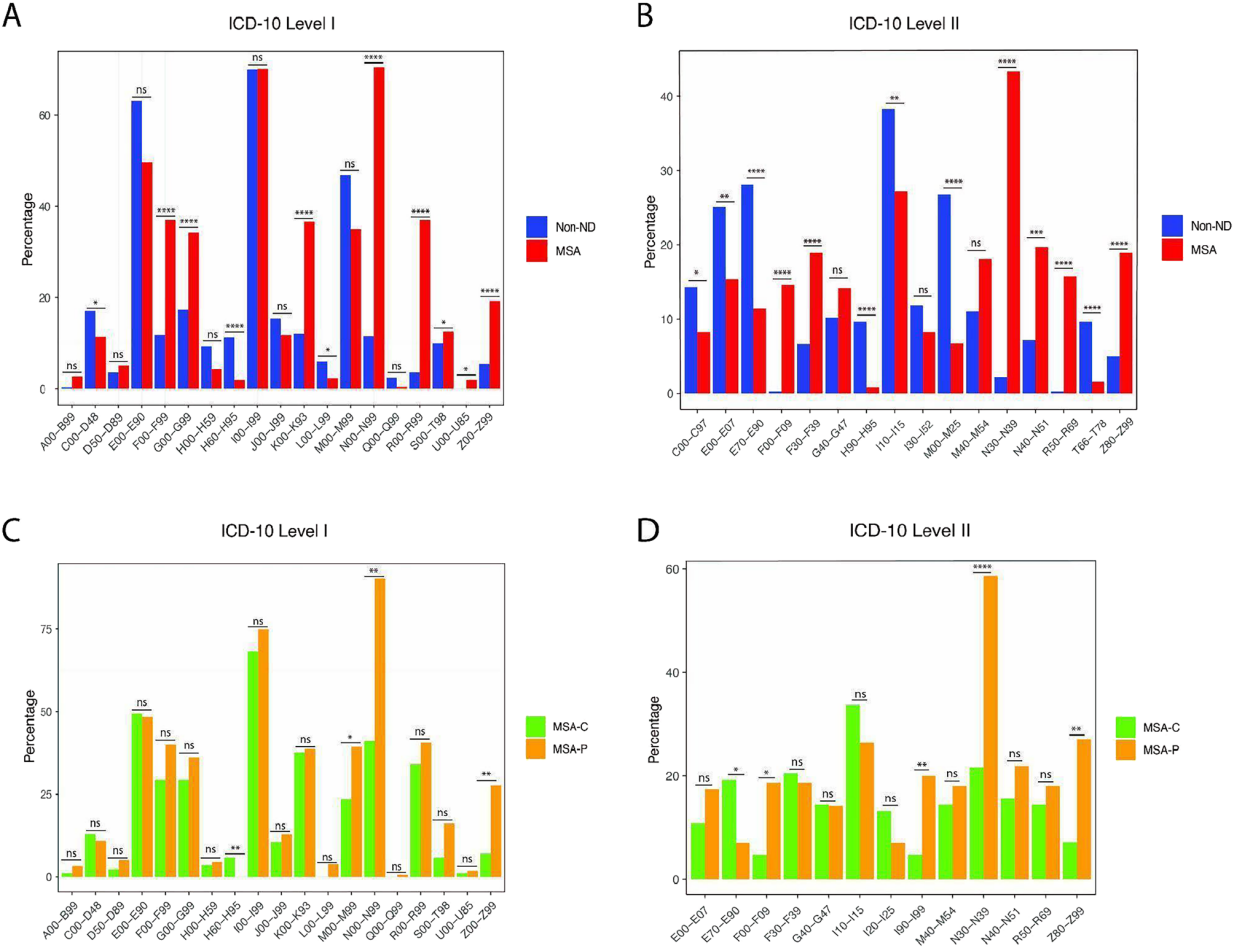
Prevalence of comorbidities according to ICD-10 classification. *p < 0.05, **p < 0.01, ***p < 0.001, ****p < 0.0001, chi-squared test. The figure illustrates the prevalence of the comorbidities on the first level (**A**, **C**) and the most common comorbidities for the MSA and the control group, respectively, on the second level (**B**, **D**) of the ICD-10 classification system. The comparison between the prevalence in MSA and control patients (A, B) as well as in MSA-C and MSA-P (**C**, **D**) is pointed out. *ICD* international classification of diseases, *MSA* multiple system atrophy, *MSA-P* multiple system atrophy with predominant parkinsonism, *MSA-C* multiple system atrophy with cerebellar ataxia, *non-ND* without neurodegenerative diseases

The total number of comorbidities was higher in MSA than in non-ND control patients (MSA: 4.4 ± 3.0, non-ND: 3.2 ± 2.2, *p* < 0.0001). Moreover, in the MSA group, a higher proportion of patients (65.0%) suffered from multimorbidity with three or more diseases than in the non-ND group (52.6%, *p* < 0.01). The prevalence of comorbidities in MSA and non-ND patients, categorized according to the main chapters of ICD-10, reveals remarkable patterns (Fig. 1). MSA patients exhibit significantly higher rates of genitourinary system diseases (ICD10: N00-N99) compared to the non-ND group (MSA: 70.5%; non-ND: 11.6%; *p* < 0.0001). Mental and behavioral disorders (ICD10: F00-F99; MSA: 37.0%, non-ND: 11.9%, *p* < 0.0001) as well as diseases of the digestive system (ICD10: K00-K93; MSA: 36.6%, non-NDs: 12.1%, *p* < 0.0001) and diseases of the nervous system (ICD10: G00-G90; MSA: 34.3%, non-ND: 17.4%, *p* < 0.001), excluding MSA itself, are also more prevalent in MSA patients compared to the non-ND control group. Notably, MSA patients are more likely to receive diagnoses related to symptoms, abnormal clinical and laboratory findings (ICD10: R00-R99; MSA: 37.0%, non-ND: 5.8%, *p* < 0.0001), as well as diagnoses related to factors influencing health status and contact with health services (ICD10: Z00-Z99; MSA: 19.3%, non-ND: 5.5%, *p* < 0.0001).

ICD10 level I: A00-B99: Certain infectious and parasitic diseases; C00-D48: Neoplasms; D50-D89: Diseases of the blood and blood-forming organs and certain disorders involving the immune mechanism; E00-E90: Endocrine, nutritional and metabolic diseases; F00-F99: Mental and behavioural disorders; G00-G90: Diseases of the nervous system; H00-H59: Diseases of the eye and adnexa; H60-H95: Diseases of the ear and mastoid process; I00-I99: Diseases of the circulatory system; J00-J90: Diseases of the respiratory system; K00-K93: Diseases of the digestive system; L00-L99: Diseases of the skin and subcutaneous tissue; M00-M99: Diseases of the musculoskeletal system and connective tissue; N00-N99: Diseases of the genitourinary system; Q00-Q99: Congenital malformations, deformations and chromosomal abnormalities; R00-R99: Symptoms and abnormal clinical and laboratory findings, not elsewhere classified; S00-T98: Injury, poisoning and certain other consequences of external causes; U00-U49: Provisional assignment of new diseases of uncertain etiology or emergency use (Covid-19 Infection); Z00-Z99: Factors influencing health status and contact with health services.

ICD10-level II: C00-C97: Malignant neoplasms; E00-E07: Disorders of the thyroid gland; E70-E90: Metabolic disorders; F00-F09: Organic, including symptomatic, mental disorders (e.g., personality and behavioural disorders); F30-F39: Mood, affective disorders; G40-G47: Episodic and paroxysmal disorders (e.g., migraine, epilepsy); G00-G47: Diseases of the nervous system (e.g., Episodic and paroxysmal disorders); H90-H95: Other disorders of the ear; I10-I15: Hypertensive disease; I20-I25: Chronic ischaemic heart disease; M00-M25: Arthropathies; M40-M54: Dorsopathies; N30-N39: Other diseases of the urinary system; N40-N51: Diseases of male genital organs; R50-R69: General symptoms and signs (e.g., fever, headache, pain, syncope, edema); T66-T78: Other and unspecified effects of external causes; Z80-Z99: Persons with potential health hazards related to family and personal history and certain conditions influencing health status (e.g., Presence of cardiac and vascular implants and grafts, Presence of other functional implants, other post-surgical states).

Examining the ICD10 level 2 data, it is clear that among the MSA patients, bladder infections and other urinary tract infections had the highest prevalence at 43.3%, while only 2.2% of the non-ND control patients were affected (ICD10:N30-N39; MSA: 43.3%, non-ND: 2.2%, *p* < 0.0001). Additionally, MSA patients showed a higher incidence of diseases related to male genital organs, such as hyperplasia of prostate (ICD10: N30-N39; MSA: 19.7%, non-ND: 7.2%, *p* < 0.001)). Consistent with this result, there is a higher prevalence of transurethral resection among MSA patients (ICD10: Z80-Z99; MSA: 19.0%, non-ND: 5.0%, *p* < 0.0001).

However, hypertensive disease is significantly less prevalent among MSA patients compared to the control group (ICD10: I10-I15; MSA: 27.2%, non-ND: 38.3%, *p* < 0.01). MSA patients showed also a decreased prevalence of disorders of the thyroid gland (ICD10: E00-E07; MSA: 15.4%, non-ND: 25.1%, *p* < 0.01) as well as metabolic disorders (ICD10: E70-E90; MSA: 11.4%, non-ND: 28.1%, *p* < 0.0001). Due to the reported association between diabetes mellitus and parkinsonism, we specifically examined the prevalence of diabetes mellitus in our analysis. Consistent with previous reports, we also observed a higher prevalence of diabetes mellitus in the MSA group compared to the non-ND control group (ICD10: E10-E14; MSA: 9.8%, non-ND: 3.9%, *p* < 0.01).

MSA patients even exhibited a lower prevalence of arthropathies (ICD10: M00-M25; MSA: 6.7%, non-ND: 26.7%, *p* < 0.0001) compared to the non-ND control group. Additionally, the prevalence of malignant neoplasms is lower among MSA patients (ICD10: C00-C97; MSA: 8.3%, non-ND: 14.3%, *p* < 0.05). There are no difference on other forms of heart disease, such as endocarditis and pericarditis, between MSA patients and the control group (ICD10: I30-I52; MSA: 8.3%, non-ND: 11.9%, *p* > 0.05).

ATC: A02B: Drugs for peptic ulcer and gastro-oesophageal reflux disease (GORD); A06A: Drugs for constipation; A11C: Vitamin A and D, incl. combination of the two; B03B: Vitamin B12 and folic acid; C07A: Beta blocking agents; C08C: Selective calcium channel blockers with mainly vascular effects; C09A: ACE inhibitors, plain; C09C: Angiotensin II receptor blockers (ARBs), plain; C10A: Lipid modifying agents, plain; G04B: Urologicals; G04C: Drugs used in benign prostatic hypertrophy; H03A: Thyroid preparations; N04B: Dopaminergic agents; N06A: Antidepressants; V06X: Other food and food supplements.

### Medication

The medication was analyzed with the help of the WHO ATC classification. Not only was the number of patients with polypharmacy significantly higher in MSA (MSA: 165 (65.0%); non-ND: 62 (17.1%), *p* < *0.0001*), but also the number of administered drugs (MSA: 6.4 ± 3.9; non-ND: 2.4 ± 2.4; *p* < *0.0001*). This trend persisted even when excluding Parkinson's medication, with MSA patients still showing a higher number of administered drugs (MSA: 5.1 ± 3.4, non-ND: 2.4 ± 2.4, *p* < 0.0001).

In line with the presented data indicating a decreased incidence of hypertensive disease among MSA patients, angiotensin II receptor blockers (ARBs) (ATC: C09C; MSA: 7.5%, non-ND: 20.4%, *p* < *0.0001*) and selective calcium channel blockers with mainly vascular effects were administered less frequently (ATC: C08C; MSA: 4.3%, non-ND: 8.8%, *p* < *0.05*) in MSA patients.

Consistent with the previous finding of a higher prevalence of diabetes mellitus in the MSA group, the proportion of patients treated with drugs used in diabetes is higher in the MSA group compared to the control group (ATC: A10; MSA: 7.5%, non-ND: 3.6%, *p* < *0.05*). However, the MSA group did not take more insulin than the non-ND group (ATC: A10A; MSA: 2.0%, non-ND: 1.4%, *p* > *0.05*).

**Fig. 2:**
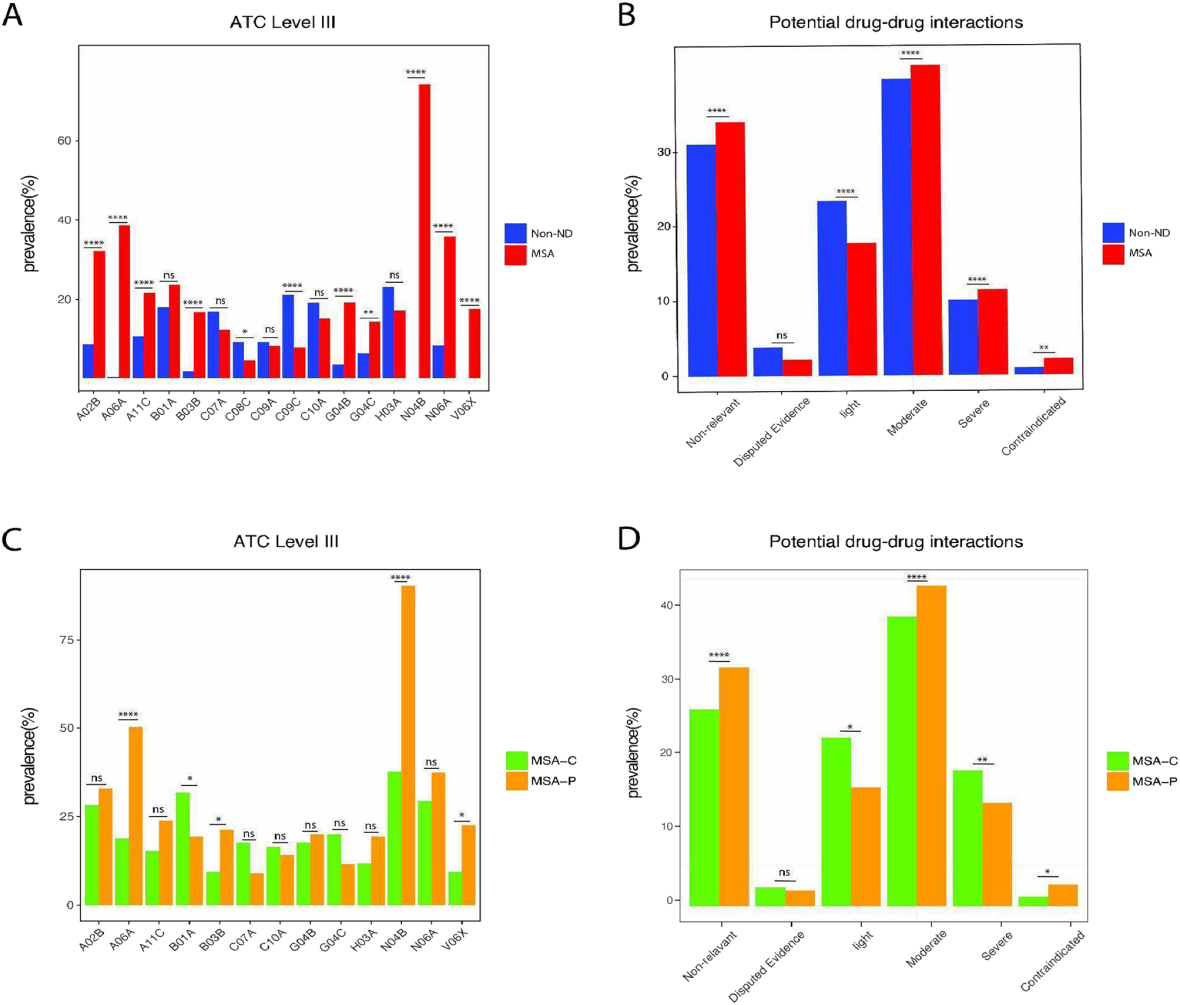
Prevalence of administered drugs according to ATC classification and potential drug-drug interactions. *p < 0.05, **p < 0.01, ***p < 0.001,****p < 0.0001, chi-squared test. The figure shows the prevalence of the most common drugs for MSA-P or for MSA-C administered on the third level of WHO ATC system (**A**, **C**) and the prevalence of pDDIs according to their severity (**B**, **D**). The comparisons between the prevalence in MSA and control patients (**A**, **B**) as well as in MSA-P and MSA-C (**C**, **D**) are indicated. *ATC* anatomical therapeutic chemical, *MSA* multiple system atrophy, *MSA-P* multiple system atrophy with predominant parkinsonism, *MSA-C* multiple system atrophy with cerebellar ataxia, *non-ND* without neurodegenerative diseases

The CDSS AiDKlinik® was used to identify pDDIs. The data are shown in Fig. 2B. MSA patients exhibited significantly more pDDIs than control patients (MSA: 2.2 ± 2.4, non-ND: 0.6 ± 1.4, *p* < 0.001). The overall distribution of the prevalence of different types of interactions in the MSA group and the control group is similar. In both groups, the prevalence of the combination of contraindicated or high-risk medications is lower than that of mild or moderate interactions. However, in the MSA group, there is a higher prevalence of severe interactions (MSA: 10.5%, non-ND: 9.2%, *p* < 0.01) and contraindicated combinations (MSA: 2.0%, non-ND: 0.9%, *p* < 0.0001). The most common severe interactions include the combination of non-steroidal anti-inflammatory drugs (NSAIDs) and serotonin-norepinephrine reuptake inhibitors (SNRIs), which can elevate the risk of gastrointestinal bleeding, the combination of aspirin and metamizole, which can practically abolish platelet aggregation, the combination of baclofen and levodopa, which can cause hallucinations, confusion, headache, nausea and worsen Parkinson's symptoms. Furthermore, combining ACE inhibitors with diuretics and NSAIDs can increase the risk of acute kidney failure. The most significant contraindicated constellation arised from the combination of amantadine and amitriptyline, which can result in QT interval prolongation. Additionally, the combination of mirtazapine and rasagiline may lead to an increased risk of serotonergic toxicity.

### Comparison of MSA subgroups

The group of all MSA patients (n = 254) was classified into two subtypes: MSA-C (n = 85, 33.5%) and MSA-P (n = 155, 61.0%). It is important to note that the phenotype for 14 MSA patients was not available. Sex and age distribution as well as the disease duration and severity did not differ between MSA-P and MSA-C patients. However, the number of total diseases was higher in MSA-P than in MSA-C patients (MSA-C: 3.6 ± 2.6, MSA-P: 5.0 ± 3.1, *p* < 0.001).

Although there was no significant difference in the percentage of most comorbidities between the two MSA subtype groups, certain disease categories showed distinctions (Fig. 1C).

MSA-P patients were found to suffer significantly more often from diseases of the musculoskeletal system and connective tissue (ICD10: M00-M99; MSA-C:23.5%, MSA-P:39.4%,* p* < 0.05), diseases of the genitourinary system (ICD10: N00-N99; MSA-C: 41.2%, MSA-P: 90.3%, *p* < 0.01), and factors influencing health status and contact with health services (ICD10: Z00-Z99; MSA-C: 7.1%, MSA-P: 27.7%, p < 0.01).

In addition, MSA-C patients had a higher prevalence of metabolic disorders (ICD10: E70-E90; MSA-C: 19.3%; MSA-P: 7.1%, p < 0.05) compared to MSA-P patients, and the prevalence of ischemic heart diseases showed a higher tendency compared to MSA-P patients (ICD10: I20-I24; MSA-C: 13.3%; MSA-P: 7.1%; *p* = *0.052*).

Regarding the medications, there were no significant differences observed in the most frequently subscribed medications between MSA-C and MSA-P patients (Fig. 2C). However, MSA-P patients were found to have a significantly higher percentage of prescriptions for dopaminergic agents compared to MSA-C patients (MSA-P: 90.3%; MSA-P: 37.7%, *p* < *0.0001*). Additionally, not only the percentage of patients prescribed with dopaminergic agents was higher among MSA-P patients, but the total levodopa equivalent dose (LED) was also significantly higher in MSA-P patients compared to MSA-C patients (MSA-P: 897.7** ± **692.5; MSA-C: 285.8** ± **479.0, *p* < *0.0001*). Levodopa is the most commonly prescribed medication among the five dopaminergic agents in both groups (Table 2).

**Table 2:** Disease-specific characteristics and dopaminergic agents in MSA patients

	MSA (n = 254)	MSA-C (n = 85)	MSA-P (n = 155)
Age, mean ± SD (min, max)	63.8 ± 8.5 (46, 82)	63.2 ± 8.6 (48, 82)	64.3 ± 8.4 (46, 82)
Sex, female (%)	49.2	41.2	51.5
Disease duration, mean ± SD, years	4.3 ± 2.2	4.8 ± 2.7	4.2 ± 2.1
Hoehn and Yahr Scale	2.0 ± 2.1	1.5 ± 2.0	2.1 ± 2.1
LED total, mean ± SD, mg	685.4 ± 698.3	285.8 ± 479.0	897.7 ± 692.5***
Levodopa (%)	77.5%	82.8%	76.8%*
Dopamine agonists (%)	16.3%	11.5%	17.1%*
MAO-inhibitors (%)	2.4%	2.7%	2.6%**
COMT-inhibitors (%)	1.2%	1.4%	1.3%**
Amantadine (%)	2.5%	2.3%	2.6%*

*ATC* anatomical therapeutic chemical, *MSA* multiple system atrophy, *MSA-P* multiple system atrophy with predominant parkinsonism, *MSA-C* multiple system atrophy with cerebellar ataxia, *LED* levodopa equivalent dose, *MAO* monoamine oxidase, *COMT* catechol-O-methyltransferase, *SD* standard deviation

^*^p < 0.05, ***p < 0.001, Mann–Whitney U test or chi-squared test

MSA patients were more frequently treated with drugs for constipation (ATC: A06A; MSA-P: 50.3%, MSA-C: 18.8%, *p* < *0.0001*). Additionally, MSA-P patients showed higher usages of vitamin B12 and folic acid (ATC: B03B; MSA-P: 21.3%, MSA-C: 9.4%, *p* < *0.05*) as well as general nutrients (ATC: V06X; MSA-P: 22.6%, MSA-C: 9.4%, *p* < *0.05*) compared to MSA-C patients.

## Discussion

In this multicenter retrospective cross-sectional study, we analyzed the medical history of 254 MSA patients. Our study reveals that MSA patients are prone to experience conditions related to the genitourinary, digestive, nervous systems, and mental health (as per ICD-10 classification). Specifically, MSA patients showed higher prevalence of cystitis, urinary tract infections and prostate hyperplasia. Polypharmacy was also more common among MSA patients, leading to more severe drug interactions. Among MSA subtypes, MSA-P patients experienced a higher prevalence of musculoskeletal and genitourinary system diseases, and they were more frequently treated with dopaminergic agents, particularly levodopa.

Although there was no difference in the age between MSA and control group, the total number of comorbidities in MSA patients were significantly higher than in the control group. Urogenital disorders, particularly cystitis and other urinary tract infections, is a notable comorbidity in MSA patients. In a study of 21 MSA patients, it was shown that urinary tract infection is at the second place for the causes of death (23.8%) in patients with MSA [12]. Another study involving 131 MSA patients also showed that 3.1% of the patients died of a urinary tract infection [13]. Although the role of urinary tract infection in mortality remains unclear in our study, the marked prevalence of comorbidity highlights the importance of focusing on prevention of urinary tract infection in MSA patients. Both overactive bladder and incomplete bladder emptying, which result from autonomic failure, in MSA patients are associated with increased risk for urinary infection [14, 15].

Actually, most patients presented with either MSA-P or MSA-C, but signs of autonomic failure are always present [3, 16]. It showed that urinary tract symptoms were present in 79.7% of the MSA patients [17]. Nonetheless, our findings indicate an elevated susceptibility to urinary system disorders, such as urinary tract infections, among patients with MSA-P rather than in patients with MSA-C. Such a comparison has not been reported so far according to our best knowledge and a further investigation into the reason should be performed.

Prostatic hyperplasia is another notable comorbidity in the MSA patients in this study. The prevalence of transurethral resection among MSA patients is also higher than in the control group. One contributing factor may be the elevated concerns from neurologists when managing MSA patients. Consequently, these patients may be promptly referred to a urologist even for subtle urological disorders. On the other side, urological symptoms associated with MSA were frequently mistaken for benign prostatic hyperplasia symptoms, resulting in unnecessary urological surgery [17]. It is not clear whether this factor contributes to the elevated incidence of prostatic hyperplasia as well.

In our study, we showed that hypertensive disease is less frequently diagnosed among MSA patients than in the control group and, consequently, MSA patients take fewer angiotensin II receptor blockers (ARBs) and selective calcium channel blockers with mainly vascular effects compared to the control group. Orthostatic hypotension and supine hypertension are core features within the spectrum of autonomic failure in MSA patients. It is encouraged to treat the supine hypertension by MSA patients with non-pharmacological measures such as heading-up tilt at night to reduce diuresis at night, increase intravascular volume, and reduce morning hypotension. In addition, there are only a few drugs with official approval available for the treatment of supine hypertension, such as clonidine, minoxidil, sildenafil, losartan and nifedipine [18].

The relationship between diabetes mellitus and Parkinson’s disease has been discussed since the early sixties [19, 20]. One hypothesis is that the two proteins, amylin and alpha-synuclein, interact in vivo and ultimately cause diabetes mellitus type 2 and Parkinson's disease [21]. Similar to Parkinson’s disease, as a subtype of synucleinopathies [21, 22], our study revealed an elevated prevalence of diabetes mellitus among MSA patients compared to the control group.

Although MSA patients experience an elevated occurrence of circulatory system disorders, it does not exceed that of the control group. This suggests that MSA itself does not emerge as a distinct risk factor for this type of disease. However, it is reported that cardiopulmonary arrest is one of the common causes of death by MSA patients [12, 13].

In our study, about 72% of all the MSA patients were treated with dopaminergic agents. MSA-P patients were found to have a significantly higher percentage of prescriptions for dopaminergic agents compared to MSA-C patients, although there is no difference on the Hoehn and Yahr scale or on the disease duration between the two subtypes. It is worth noting that a poor response to levodopa is actually a defining feature of parkinsonism in MSA [23]. However, individuals with MSA-P might exhibit a transit favorable therapeutic reaction to levodopa, which might explain the more frequent administration of dopaminergic agents in this group [24]. The most widely prescribed dopaminergic agent in both subtypes in our study was levodopa, followed by dopamine agonists, monoamine oxidase inhibitors (MAOIs), amantadine, catechol-O-methyl-transferase (COMT) inhibitors and anticholinergics. This is in line with the suggestion that dopaminergic substitution by levodopa as first choice and dopamine agonists as well as non-dopaminergic drugs (such as amantadine) second line [3]. However, after examining the pDDIs, quite a few of severe interactions and contraindicated combinations were related to these kinds of drugs. This underscores the importance of carefully considering and managing drug interactions when treating patients with MSA and related conditions.

Our study offered several limitations. Firstly, the quality of the data depends on the accuracy and completeness of medical records, which can vary. Secondly, we analyzed all documented diseases and operations; however, only the medication at the last visit could bewas analyzed.. Thirdly, due to the limited sample size givenbecause of the rarity of the disease, the statistical results for the less common factors might not be accurate.

In summary, this study represents to our knowledge the first comprehensive examination of comorbidities and co-medication in MSA patients. Despite the rarity of the disease, our large collection of 254 MSA patients yields a compelling sample size. Apart from some diseases that are already prevalent in the general population such as diseases of the circulatory system, the primary comorbidities for patients with MSA relate mainly to the autonomic disorder, instead of the MSA-associated motor symptoms. This emphasizes that effective management of MSA should not only address the motor symptoms of the disease, but also avoid underestimating the importance of treating autonomic dysfunction.

## Data Availability

The data supporting the findings of this study are available from the corresponding author Dr. Stephan Greten upon reasonable request.
